# The complete mitochondrial genome of the Tamarisk gerbil, *Meriones tamariscinus* (Rodentia: Muridae)

**DOI:** 10.1080/23802359.2016.1247662

**Published:** 2017-01-04

**Authors:** Chang-Long Li, Cun-Long Wang, Shun-Sheng Yan, Xin-Ru Chen, Meng Guo, Xue-Yun Huo, Zhen-Kun Li, Xiao-Yan Du, Zhen-Wen Chen

**Affiliations:** a School of Basic Medical Science, Capital Medical University, Beijing, China;; b The Center for Disease Control and Prevention of Xinjiang Uygur Autonomous Region, Urumqi, China

**Keywords:** Phylogenetic analysis, Tamarisk jird, mitogenome

## Abstract

The complete mitochondrial genome of the Tamarisk jird, *Meriones tamariscinus*, was sequenced. The 16,389bp genome contains 37 genes, typical for rodent mitogenomes, including 22 tRNA genes, 2 rRNA genes, and 13 protein-coding genes. The total GC content of the mitochondrial genome is 36.8%, with a base composition of 34.0% A, 24.5% C, 12.3% G, and 29.2% T. The phylogenetic analysis showed that *M. tamariscinus* was classified in the genus *Meriones*, Muridae.

The Tamarisk jird (*Meriones tamariscinus*) is a rodent that inhabits desert and semi-desert areas. The animal was collected from Khorgos (44°7′39″N, 80°24′13E), China, and was identified by morphology and DNA sequence (Gu et al. 2011). The complete mitochondrial genome of *M. tamariscinus* (GenBank accession no. KX688104) was sequenced by using long PCR and Sanger sequencing. The circular mitochondrial genome was 13,689 bp long and contained 37 genes (2 rRNA genes, 22 tRNA genes, and 13 protein-coding genes) that are typically found in rodent mitochondrial genomes (Table 1) (Jiang et al. 2012). The base composition was 29.2% T, 24.5% C, 34.9% A and 12.3% G. The overall disproportional A + T content was 63.2%, which was higher than any of the three previously studied *Meriones* species (*M. unguiculatus*, 63%; *M. libycus*, 61.6%; and *M. meridianus*, 62.7%)(Luo & Liao 2016a, 2016b). ATG was used as the start codon of eight protein-coding genes, while ATA was used by *ND1*, GTG was used by *ND4L*, and ATT was used by *ND2*, *ND3,* and *ND5*. The unusual start codons ATA and ATT have also been observed in other mammals (Jiang et al. 2012). The coding regions of the genes *Cox1*, *Cox2*, *ATPase8*, *ATPase6*, *ND3*, *ND4L,* and *ND5* were terminated by TAA, while *ND6* was terminated by TAG. Five incomplete stop codons T(––) were found in the *ND1, ND2, Cox3, ND4,* and cyt*b* genes. Incomplete stop codons have been observed in many other mammals (Peng et al. 2007). Several pairs of genes overlapped: *ATPase8* and *ATPase6*, *ATPase6* and *Cox3*, and *ND4L* and *ND4* (Table 1). Two rRNA genes (*12S rRNA* and *16S rRNA*) were identified via DOGMA with default settings and refined through alignment to homologous genes in other mitochondrial genomes. The 12S *rRNA* gene was 951 bp in length, and the 16S *rRNA* gene was 1572 bp in length. These genes had 61.5% and 63.8% A + T content, respectively. They were flanked by *tRNA^Phe^* and *tRNA^Leu^* and separated by *tRNA^Val^*. The length of *M. tamariscinus* tRNA genes ranged from 59 to 75 bp. Of the 22 tRNA genes, 21 could fold into a cloverleaf secondary structure. The *tRNA^ser(AGN)^*was only 59 bp in length and was missing the dihydrouridine stem and loop. This loss is common among vertebrates (Zhong et al. 2010). The putative origin of replication for the light strand (O_L_) is located in the WANCY cluster between *tRNA^Asn^* and *tRNA^Cys^*. The concatenated nucleotide sequence of the 12 protein-coding genes encoded on the H-strand from 26 available mitochondrial genomes and *M. tamariscinus* mitogenome were used to reconstruct the phylogenetic relationships using neighbour-joining (NJ), maximum-likelihood (ML), and minimum evolution(ME) methods (Ito et al. 2010). The phylogenetic methods produced the same tree topology in the branching patterns. The monophyly of the *Meriones* clade received relatively strong support (Figure 1; BP = 100%). The phylogenetic analysis showed *Rattus* forming a clade with *Mus*, and this clade was closer to the *Meriones* clade than was the other clade. This finding demonstrates that *Meriones,* including *M. libycus and M. meridianus,* should be placed in *Muridae*.

**Figure 1.**
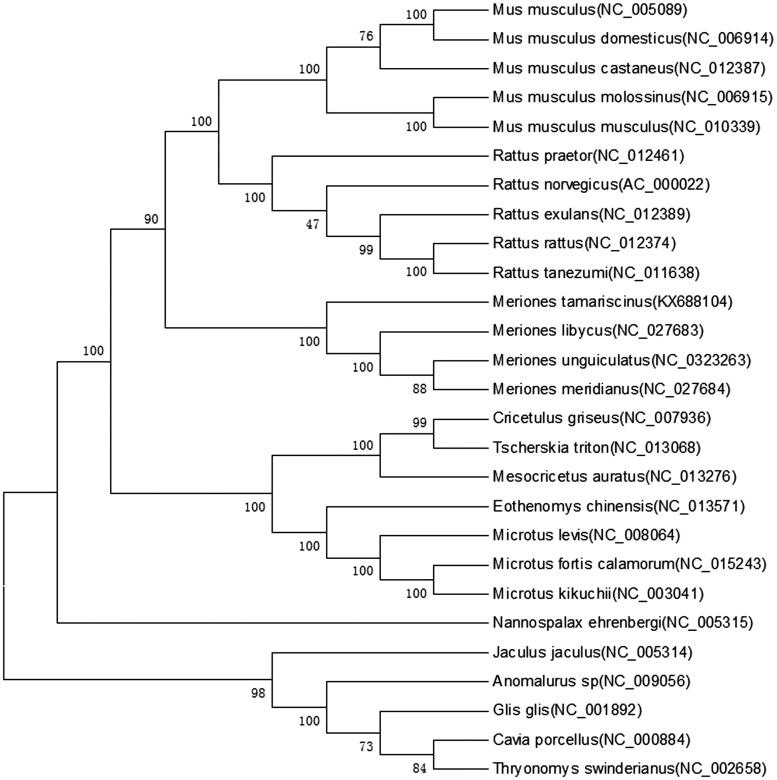
A maximum-likelihood tree of *M. tamariscinus* and 26 rodents inferred from 12 mitochondrial genes in H-strand. Percentage bootstrap values are shown on interior branches with 1000 replicates for the ML method. GenBank accession numbers are indicated in brackets.

**Table 1.** Elements and base composition of *M. tamariscinus* mitochondrial genome.

					Base composition (%)				
Gene	Position	Size (bp)	Start	Stop	T	C	A	G	Strand
*tRNA^*Phe*^*	1–69	69			23.2	21.7	39.1	15.9	H
*12S RNA*	70–1020	951			25.1	21.1	36.4	17.1	H
*tRNA^*Val*^*	1021–1087	67			22.4	22.4	40.3	14.9	H
*16S RNA*	1086–2657	1572			25.5	19.5	38.3	16.7	H
*tRNA^*Leu1*^*	2660–2734	75			26.7	17.3	34.7	17.3	H
*NADH1*	2735–3689	955	ATA	T––	31.6	27.2	29.4	11.7	H
*tRNA^*Ile*^*	3690–3757	68			32.4	16.2	39.7	16.2	H
*tRNA^*Gln*^*	3826–3755	72			26.4	29.2	30.6	13.9	L
*tRNA^*Met*^*	3838–3906	69			18.8	31.9	30.4	18.8	H
*NADH2*	3910–4948	1039	ATT	T––	30.8	25.5	36.1	7.6	H
*tRNA^*Trp*^*	4947–5011	65			24.6	20.0	35.4	11.6	H
*tRNA^*Ala*^*	5082–5014	69			30.4	23.2	34.8	30.4	L
*tRNA^*Asn*^*	5158–5088	71			28.2	26.8	29.6	28.2	L
*tRNA^*Cys*^*	5255–5189	67			35.8	19.4	31.3	13.4	L
*tRNA^*Tyr*^*	5343–5276	68			30.9	23.5	30.9	22.7	L
*Cox1*	5345–6889	1545	ATG	TAA	30.1	24.6	30.2	15.1	H
*tRNA^*Ser1*^*	6955–6887	69			26.1	27.5	31.9	14.5	L
*tRNA^*Asp*^*	6959–7027	69			36.2	8.7	44.9	10.1	H
*Cox2*	7029–7715	687	ATG	TAA	27.4	25.3	34.2	13.1	H
*tRNA^*Lys*^*	7716–7780	65			30.8	16.9	36.9	15.4	H
*ATPase 8*	7782–7985	204	ATG	TAA	27.5	24	42.2	6.4	H
*ATPase 6*	7944–8624	681	ATG	TAA	28.9	28.3	32.9	9.8	H
*Cox3*	8624–9407	784	ATG	T––	29.5	26.1	30.9	13.5	H
*tRNA^*Gly*^*	9408–9475	68			32.4	14.7	38.2	14.7	H
*NADH3*	9476–9823	348	ATT	TAA	35.3	24.4	29.6	10.6	H
*tRNA^*Arg*^*	9828–9895	68			39.7	13.2	36.8	10.3	H
*NADH4L*	9897–10,193	297	GTG	TAA	31.6	24.9	31	12.5	H
*NADH4*	10,187–11,564	1378	ATG	T––	29.8	26.5	34.5	9.3	H
*tRNA^*His*^*	11,565–11,633	69			34.8	13	39.1	13	H
*tRNA^*Ser2*^*	11,634–11,692	59			25.4	22	35.6	16.9	H
*tRNA^*Leu*^*	11,693–11,761	68			30.4	17.4	37.7	14.5	H
*NADH5*	11,762–13,573	1812	ATT	TAA	30.5	26.4	33.7	9.4	H
*NADH6*	13,575–14,093	519	ATG	TAG	21.4	27.7	43.4	7.5	L
*tRNA^*Glu*^*	14,162–14,094	69			31.6	25.2	30.6	12.6	L
*Cyt b*	14,167–15,310	1144	ATG	T––	31.6	25.2	30.6	12.6	H
*tRNA^*Thr*^*	15,311–15,377	67			31.3	14.9	35.8	17.9	H
*tRNA^*Pro*^*	15,378–15,445	68			19.1	27.9	36.8	16.2	L
*CR*	15,446–16,389	944			30.9	23.8	34.2	11	H
